# Transient cognitive impairment in the acute phase of stroke – prevalence, risk factors and influence on long-term prognosis in population of patients with stroke (research study – part of the PROPOLIS study)

**DOI:** 10.1186/s12883-023-03120-x

**Published:** 2023-02-17

**Authors:** Jakub Droś, Katarzyna Kowalska, Paulina Pasińska, Aleksandra Klimkowicz-Mrowiec

**Affiliations:** 1 Doctoral School in Medical and Health Sciences, Jagiellonian University Medical College, Kraków, Poland; 2 St. Louis Regional Specialized Children’s Hospital, Kraków, Poland; 3 Department of Medical Didactics, Faculty of Medicine, Jagiellonian University Medical College, Kraków, Poland; 4 Department of Internal Medicine and Gerontology, Faculty of Medicine, Jagiellonian University Medical College, Kraków, Poland

**Keywords:** Stroke, Cognition disorders, Dementia

## Abstract

**Background:**

Cognitive impairment is a common complication of the acute phase of stroke, which can be transient and resolve while still in the hospital. This study evaluated the prevalence and risk factors for transient cognitive impairment and their impact on long-term prognosis in a population of acute-phase stroke patients.

**Methods:**

Consecutive patients admitted to a stroke unit with acute stroke or transient ischemic attack were screened twice for cognitive impairment using the parallel version of Montreal Cognitive Assessment: the first time between the first and third day and the second time between the fourth and seventh day of hospitalization. If the second test score increased by two or more points, transient cognitive impairment was diagnosed. Patients were scheduled for follow-up visits three and 12 months after stroke. Outcome assessment included place of discharge, current functional status, dementia, or death.

**Results:**

Four hundred forty-seven patients were included in the study, 234 (52.35%) were diagnosed with transient cognitive impairment. Delirium was the only independent risk factor for transient cognitive impairment (OR 2.417, 95%CI 1.096–5.333, *p* = 0.029). In the analysis of effects on three- and twelve-month prognosis, patients with transient cognitive impairment had a lower risk of hospital or institution stay 3 months after stroke compared with patients with permanent cognitive impairment (OR 0.396, 95%CI 0.217–0.723, *p* = 0.003). There was no significant effect on mortality, disability or risk of dementia.

**Conclusions:**

Transient cognitive impairment, which often occurs in the acute phase of stroke, does not increase the risk of long-term complications.

**Supplementary Information:**

The online version contains supplementary material available at 10.1186/s12883-023-03120-x.

## Background

Cognitive impairment (CI) is a common complication of stroke, leading to increased risk of mortality, disability and institutionalization [1–3]. Cognitive deficits often develop in the acute phase of stroke and affect between 59 and 88% of stroke survivors [4–6]. CI identified early after stroke has been shown to predict functional outcomes and subsequent cognitive deficits in the long-term [6, 7]. In acute stroke survivors, CI may be transient and resolve during hospitalization [8].

Delirium, a transient condition of impaired attention and consciousness, is one of the most common complication of acute hospital admissions, leading to increased post-discharge mortality and institutionalization [9]. Post-stroke delirium occurs in about a fourth of acute stroke patients [10], and is similarly associated with worse outcomes, increasing the risk of death, functional disability and nursing home placement [11, 12]. Delirium and transient CI seem to be distinct disease entities, but their symptoms may overlap being difficult to differentiate in the clinical practice. Post-stroke delirium has been extensively described in the literature, but so far, the prevalence and impact of transient CI, which occurs and resolves during hospitalization, on subsequent prognosis of stroke patients has not been studied.

Therefore, the aim of this study was to evaluate the prevalence and factors associated with transient CI that occurs and resolves during the acute phase of stroke, and to investigate the impact of these transient cognitive deficits on three-month and one-year prognosis.

## Methods

This study was part of a large single-center PRospective Observational POLIsh Study on post-stroke delirium (“PROPOLIS”) conducted at Jagiellonian University Medical College in Kraków, Poland [13]. All procedures performed in this study involving human participants were in accordance with the ethical standards of the institutional and national research committee and with the 1964 Helsinki declaration and its later amendments. Informed written consent was provided by each participant or a caregiver. The study protocol was approved by the Bioethics Committee of Jagiellonian University (KBET/63/B/2014).

Consecutive patients with acute stroke or transient ischemic attack (TIA) admitted to the Stroke Unit of the University Hospital in Kraków, fulfilling the inclusion criteria (patients > 18 years of age, admitted within 48 hours of the first stroke symptoms, Polish-speaking), and in whom cognitive performance could be assessed twice during the hospital stay were included in the study. Patients with coma, brain tumor, alcohol withdrawal delirium, cerebral venous thrombosis, subarachnoid hemorrhage, trauma, disease with life expectancy < 12 months and those who were not able to be evaluated with MoCA, or did not consent for the cognitive examination in hospital were excluded from the study. Cognitive status was assessed using two parallel versions of the Montreal Cognitive Assessment (MoCA) [14]; the first time between the first and third day and the second time between the fourth and seventh day after stroke. The time between the two assessments was three to four days in each case. A trained psychologist was responsible for cognitive assessment, and the senior neurologist/neuropsychologist evaluated all data.

Two MoCA test scores were compared in each case. If the second test score increased by two or more points, transient CI was diagnosed. When the second cognitive assessment showed a score two or more points lower, patients were defined “cognitively impaired”. If the score did not change or the MoCA score difference was less than two points, patients were classified as “cognitively stable”.

Data were also collected regarding socio-demographic factors (age, gender, education), medical history (comorbidities, medications, infections, biochemical disturbances), and stroke-related features (type of stroke, severity, stroke symptoms). The Cumulative Illness Rating Scale (CIRS) was used as a general indicator of health status [15]. On admission, information was obtained from a spouse or a caregiver regarding pre-stroke cognitive functioning on the Polish version of Informant Questionnaire on Cognitive Decline in the Elderly (IQCODE) [16, 17]. Disability prior to admission was assessed by the modified Rankin Scale (mRS) [18].

All patients had neuroimaging (computed tomography/magnetic resonance) performed during admission. The severity of clinical deficit was graded by the National Institutes of Health Stroke Scale (NIHSS) [19], upon admission. Furthermore, the subtype of ischemic stroke was evaluated using the Trial of ORG 10172 in Acute Stroke Treatment (TOAST) classification [20]. Data regarding aphasia, neglect or vision deficits were also collected.

Every day, from admission to the seventh day of hospitalization, patients were tested for delirium using the abbreviated version of Confusion Assessment Method (bCAM). In patients with motor aphasia or in those who could not communicate for other reasons, the Intensive Care Unit version (CAM-ICU) was used [21, 22].

All patients discharged from the hospital were scheduled for follow-up visits three and 12 months after stroke with a tolerance of up to 4 days each way. If any patient failed to show up for an appointment, they were contacted the same day and interviewed by telephone. In cases where patients could not be interviewed, the patient’s caregiver was contacted and interviewed. Outcome assessment included place of discharge (home, rehabilitation hospital, or long-term institution), current functional status (mRS), or death. Mortality data were collected when a researcher was reliably informed of the participant’s death, usually by a close informant from the participant’s household. At follow-up outpatient visits, patients underwent neuropsychological testing. The details of cognitive evaluation were described elsewhere [23]. Adverse prognosis was defined as the occurrence of any of the following: death, increase in mRS score by ≥1 (between pre-hospital and follow-up visit), hospital or institution stay, and dementia.

Statistical analysis was performed using Statistica 13.3 software (StatSoft®, Poland). Qualitative variables were compared using the chi-squared test with or without Yates’ correction, while quantitative variables were compared with the Mann-Whitney U test, or in case of more than two groups with Kruskal–Wallis H test (one-way ANOVA on ranks), due to non-normal data distribution. In search of independent risk factors for the incidence of transient CI compared to ‘cognitively stable’ cases, univariate logistic regression models including considerable demographic and clinical factors, and then a multivariate logistic regression model, adjusted for age, gender, years of education, CIRS score, NIHSS score and other relevant variables at *P*-value < 0.01, were performed. To analyze the influence of transient CI on further prognosis, its predictive values on death, increase in mRS score of ≥1, hospital or institution stay, and dementia were calculated. Patients with transient CI were separately compared to cases with stable and decreasing MoCA score. Each outcome analysis included first multivariate logistic regression model adjusted for age, gender, years of education, CIRS score and NIHSS score, and the second – adjusted also for the incidence of in-hospital delirium. Continuous values were presented as medians with interquartile ranges (IQRs), and predictive values – as odds ratios (ORs) with 95% confidence intervals (CIs). *P*-values < 0.05 were considered statistically significant.

## Results

Of the 750 consecutive stroke patients initially enrolled in the study, 447 (59.60%) were eventually included, having both MoCAs performed in hospital. Differences in socio-demographic, medical and stroke-related factors between included and excluded cases are presented in Table 1. There were more men in the included group, patients were younger and with longer education. The etiology of stroke was more often cardioembolic and less often small vessel occlusion in the included patients compared to excluded cases. Stroke affected the right hemisphere of the brain or posterior vasculature more often than the left hemisphere in the included patients. Patients included in the study also had lower CIRS, NIHSS, pre-hospital mRS and IQCODE scores and lower CRP levels. Subjects included in the study were less likely to be diagnosed with aphasia, visual impairment and delirium during their hospital stay. Finally, there was a lower in-hospital mortality in the group included in the study.

**Table 1:** Comparison of socio-demographic, medical and stroke-related factors between included and excluded patients into the study

Variable	Data	Included patients (***n*** = 447)	Excluded patients (***n*** = 303)	***P***-value
Male gender^a^	750	230/447 (51.45%)	122/303 (40.26%)	0.003
Age [years]^b^	750	69 (61–79)	78 (68–84)	< 0.001
BMI [kg/m^2^]^b^	730	26.67 (23.88–29.74)	26.44 (23.80–30.00)	0.757
Length of education [years]^b^	658	11 (10–13)	11 (8–13)	0.038
Hemorrhagic stroke^a^	750	28/447 (6.26%)	24/303 (7.92%)	0.381
TOAST classification				
large-artery atherosclerosis^a^	650	41/401 (10.22%)	32/249 (12.85%)	0.302
cardioembolism^a^	650	24/401 (5.99%)	2/249 (0.80%)	0.001
small-vessel occlusion^a^	650	125/401 (31.17%)	117/249 (46.99%)	< 0.001
other determined etiology^a^	650	207/401 (51.62%)	96/249 (38.55%)	0.001
undetermined etiology^a^	650	4/401 (1.00%)	2/249 (0.80%)	0.801
Side of stroke				
right hemisphere^a^	750	208/447 (46.53%)	90/303 (29.70%)	< 0.001
left hemisphere^a^	750	170/447 (38.03%)	194/303 (64.03%)	< 0.001
posterior part^a^	750	61/447 (13.65%)	12/303 (3.96%)	< 0.001
more than one localization^a^	750	8/447 (1.79%)	7/303 (2.31%)	0.617
rt-Pa treatment^a^	750	108/447 (24.16%)	80/303 (26.40%)	0.487
Thrombectomy^a^	750	19/447 (4.25%)	13/303 (4.29%)	0.979
CIRS, total score^b^	747	8 (5–12)	10 (7–14)	< 0.001
Aphasia in hospital^a^	750	63/447 (14.09%)	192/303 (63.37%)	< 0.001
Neglect in hospital^a^	750	54/447 (12.08%)	40/303 (13.20%)	0.649
Vision deficits in hospital^a^	750	123/447 (27.52%)	175/303 (57.76%)	< 0.001
Delirium in hospital^a^	750	77/447 (17.23%)	126/303 (41.58%)	< 0.001
Death in hospital^a^	750	2/447 (0.45%)	66/303 (21.78%)	< 0.001
NIHSS at admission^b^	750	4 (2–9)	14 (5–20)	< 0.001
Pre-hospital mRS^b^	749	0 (0–0)	0 (0–2)	< 0.001
Pre-hospital IQCODE^b^	609	78 (78–81)	79 (78–90)	< 0.001
CRP level in hospital [mg/l]^b^	729	4.74 (1.99–12.31)	12.90 (4.09–38.71)	< 0.001

^a^n (%)

^b^median (IQR)

Of the 447 patients, we classified 234 (52.35%) as having transient CI, 135 (30.20%) as “cognitively stable” and 78 (17.45%) as “cognitively impaired”. Comparison in socio-demographic, medical and stroke-related factors between these three groups is presented in Table S1 in the supplementary material. The patients differed in terms of age, length of education, frequency of atrial fibrillation, CIRS, neglect and vision deficits in hospital, pre-hospital mRS and CRP level. Patients cognitively impaired were older, less educated, more often had higher CIRS score and pre-hospital, mRS, atrial fibrillation, vision deficits, neglect, higher CRP levels and delirium at the hospital,

Significant predictors of transient CI after stroke compared to stable cognitive status in the univariate logistic regression analysis are shown in Table 2. Older and less educated patients and patients with in-hospital delirium had a higher risk of transient CI. In the multivariate logistic regression model, only delirium remained an independent risk factor for transient CI (OR 2.417, 95%CI 1.096–5.333, *p* = 0.029).

**Table 2:** Analysis of risk factors for post-stroke transient cognitive impairment (CI) compared to patients with stable MoCA score in the univariate logistic regression model

Variable	Data	Transient CI (***n*** = 234)	Cognitively stable (***n*** = 135)	OR (95%CI)	***P***-value
**First MoCA score**^b^	369	**19 (13–23)**	**24 (19–26)**	N/A	< 0.001
**Second MoCA score**^b^	369	**24 (20–27)**	**23 (19–27)**	N/A	0.797
Male gender^a^	369	124/234 (52.99%)	73/135 (54.07%)	0.957 (0.626–1.464)	0.841
Age [years]^b^	369	68.5 (62–79)	65 (58–77)	1.019 (1.003–1.036)	0.019
BMI [kg/m^2^]^b^	363	26.73 (24.07–30.08)	25.78 (23.44–29.38)	1.023 (0.978–1.071)	0.316
Length of education [years]^b^	362	11 (8–13)	12 (10–14)	0.928 (0.868–0.991)	0.026
Hemorrhagic stroke^a^	369	14/234 (5.98%)	8/135 (5.93%)	1.010 (0.412–2.474)	0.982
TOAST classification					
Large-artery atherosclerosis^a^	332	24/211 (11.37%)	10/121 (8.26%)	1.425 (0.657–3.090)	0.370
Cardioembolism^a^	332	12/211 (5.69%)	11/121 (9.09%)	0.603 (0.258–1.412)	0.244
Small-vessel occlusion^a^	332	66/211 (32.28%)	33/121 (27.27%)	1.214 (0.740–1.991)	0.443
Other determined etiology^a^	332	109/211 (51.66%)	64/121 (52.89%)	0.952 (0.608–1.489)	0.829
Undetermined etiology^a^	332	0/211 (0%)	3/121 (2.48%)	–	–
Side of stroke					
Right hemisphere^a^	369	106/234 (45.30%)	67/135 (49.63%)	0.840 (0.550–1.285)	0.422
Left hemisphere^a^	369	94/234 (40.17%)	48/135 (35.56%)	1.217 (0.785–1.887)	0.380
Posterior part^a^	369	30/234 (12.82%)	19/135 (14.07%)	0.898 (0.484–1.666)	0.733
More than one localization^a^	369	4/234 (1.71%)	1/135 (0.74%)	2.330 (0.258–21.067)	0.451
rt-Pa treatment^a^	369	62/234 (26.50%)	24/135 (17.78%)	1.667 (0.893–2.827)	0.058
Thrombectomy^a^	369	10/234 (4.27%)	5/135 (3.70%)	1.161 (0.388–3.470)	0.790
Medical history					
Hypertension^a^	369	162/234 (69.23%)	92/135 (68.15%)	1.052 (0.667–1.660)	0.829
Diabetes^a^	369	66/234 (28.21%)	36/135 (26.67%)	1.080 (0.671–1.739)	0.750
Atrial fibrillation^a^	369	50/234 (21.37%)	18/135 (13.33%)	1.766 (0.983–3.175)	0.057
Myocardial infraction^a^	369	30/234 (21.82%)	18/135 (13.33%)	0.956 (0.511–1.789)	0.889
PCI or CABG^a^	369	16/234 (6.84%)	12/135 (8.89%)	0.752 (0.345–1.642)	0.475
Smoking – ever^a^	368	18/233 (50.64%)	73/135 (54.07%)	0.871 (0.570–1.333)	0.526
Previous stroke or TIA^a^	367	41/233 (17.60%)	24/134 (17.91%)	0.979 (0.562–1.706)	0.940
CIRS, total score^b^	369	7 (5–11)	8 (4–12)	1.007 (0.962–1.054)	0.774
Aphasia in hospital^a^	369	35/234 (14.96%)	14/135 (10.37%)	1.520 (0.786–2.940)	0.213
Neglect in hospital^a^	369	22/234 (9.40%)	15/135 (11.11%)	0.830 (0.415–1.661)	0.599
Vision deficits in hospital^a^	369	64/234 (27.35%)	29/135 (21.48%)	1.376 (0.834–2.272)	0.212
Delirium in hospital^a^	369	41/234 (17.52%)	10/135 (7.41%)	2.655 (1.284–.5.494)	0.009 *
NIHSS at admission^b^	369	4 (2–9)	3 (2–7)	1.030 (0.986–1.077)	0.188
Pre-hospital mRS^b^	369	0 (0–0)	0 (0–0)	1.006 (0.812–1.247)	0.953
Pre-hospital IQCODE^b^	312	78 (78–82)	78 (78–80)	1.024 (0.987–1.063)	0.199
CRP level in hospital [mg/l]^b^	356	4.12 (1.88–11.40)	4.57 (1.78–11.96)	1.002 (0.996–1.007)	0.574

*significant in the multivariate logistic regression model (OR 2.417, 95%CI 1.096–5.333, *p* = 0.029)

^a^n (%)

^b^median (IQR)

Out of the 447 patients at the three-month follow-up, 297 attended face-to-face visits, 106 were interviewed by telephone or the interviews were collected from the caregivers, 23 died and there was no contact to 21 cases. Then, out of the 424 remaining patients at the twelve-month follow-up, 213 attended face-to-face visits, 157 were interviewed by telephone or the interviews were collected from the caregivers, 23 died and there was no contact to 31 cases.

The effect of transient CI on further prognosis 3 months and 1 year after stroke was compared separately for “cognitively stable” and “cognitively impaired” cases. The results are presented in Tables 3, 4, 5 and 6. Patients with transient CI had a lower risk for hospital or institution stay 3 months after stroke compared to “cognitively impaired” (OR 0.396, 95%CI 0.217–0.723, *p* = 0.003) and this was the only significant association in the multivariate regression models adjusted for age, gender, years of education, CIRS score and NIHSS score. Adjusting for the incidence of in-hospital delirium did not influence the results.

**Table 3:** Influence of post-stroke transient cognitive impairment (CI) compared to ‘cognitively stable’ patients on three-month prognosis

Variable	Data	Incidence, n (%)		Univariate regression model		Multivariate regression model ^b^		Multivariate regression model ^c^	
		Transient CI (***n*** = 234)	Cognitively stable (***n*** = 135)	OR (95%CI)	***P***-value	OR (95%CI)	***P***-value	OR (95%CI)	***P***-value
Mortality	363	10/231 (4.33%)	3/132 (2.27%)	1.946 (0.526–7.199)	0.319	1.625 (0.415–6.364)	0.486	1.530 (0.387–6.047)	0.544
Increase in mRS of ≥1	350	137/224 (61.16%)	79/126 (62.70%)	0.937 (0.597–1.469)	0.776	0.765 (0.468–1.248)	0.283	0.700 (0.427–1.149)	0.158
Hospital or institution stay	352	54/225 (24.00%)	34/127 (26.77%)	0.864 (0.525–1.421)	0.564	0.838 (0.494–1.422)	0.512	0.800 (0.469–1.366)	0.414
Dementia	270	45/174 (25.86%)	21/96 (21.88%)	1.246 (0.690–2.250)	0.466	1.030 (0.525–2.021)	0.931	0.965 (0.483–1.927)	0.919
WORSE OUTCOME^a^	323	154/208 (74.04%)	89/115 (77.39%)	0.833 (0.488–1.424)	0.504	0.624 (0.347–1.125)	0.117	0.569 (0.314–1.030)	0.062

^a^death *or* increase in mRS of ≥1 *or* hospital/institution stay *or* dementia

^b^adjusted for age, gender, years of education, CIRS score and NIHSS score

^c^adjusted for age, gender, years of education, CIRS score, NIHSS score and incidence of in-hospital delirium

**Table 4:** Influence of post-stroke transient cognitive impairment (CI) compared to ‘cognitively impaired’ patients on three-month prognosis

Variable	Data	Incidence, n (%)		Univariate regression model		Multivariate regression model ^b^		Multivariate regression model ^c^	
		Transient CI (***n*** = 234)	Cognitively impaired (***n*** = 78)	OR (95%CI)	***P***-value	OR (95%CI)	***P***-value	OR (95%CI)	***P***-value
Mortality	306	10/231 (4.33%)	10/75 (13.33%)	0.294 (0.117–0.737)	0.009	0.410 (0.152–1.110)	0.079	0.454 (0.163–1.264)	0.131
Increase in mRS of ≥1	294	137/224 (61.16%)	53/70 (75.71%)	0.505 (0.275–0.928)	0.028	0.626 (0.328–1.195)	0.155	0.664 (0.343–1.285)	0.224
Hospital or institution stay	295	54/225 (24.00%)	31/70 (44.29%)	0.397 (0.226–0.697)	0.001	0.396 (0.217–0.723)	0.003	0.410 (0.223–0.753)	0.004
Dementia	226	45/174 (25.86%)	16/52 (30.77%)	0.785 (0.398–1.549)	0.485	0.963 (0.452–2.052)	0.923	1.116 (0.516–2.633)	0.713
WORSE OUTCOME ^a^	276	154/208 (74.04%)	60/68 (88.24%)	0.380 (0.171–0.846)	0.018	0.470 (0.203–1.091)	0.079	0.500 (0.212–1.177)	0.112

^a^death *or* increase in mRS of ≥1 *or* hospital/institution stay *or* dementia

^b^adjusted for age, gender, years of education, CIRS score and NIHSS score

^c^adjusted for age, gender, years of education, CIRS score, NIHSS score and incidence of in-hospital delirium

**Table 5:** Influence of post-stroke transient cognitive impairment (CI) compared to ‘cognitively stable’ patients on one-year prognosis

Variable	Data	Incidence, n (%)		Univariate regression model		Multivariate regression model^b^		Multivariate regression model^c^	
		Transient CI (***n*** = 234)	Cognitively stable (***n*** = 135)	OR (95%CI)	***P***-value	OR (95%CI)	***P***-value	OR (95%CI)	***P***-value
Mortality	344	21/215 (9.77%)	9/129 (6.98%)	1.443 (0.640–3.255)	0.377	1.187 (0.494–2.851)	0.702	1.085 (0.445–2.645)	0.858
Increase in mRS of ≥1	300	43/185 (23.24%)	33/115 (28.70%)	1.143 (0.731–1.788)	0.558	1.041 (0.646–1.675)	0.870	1.010 (0.626–1.631)	0.966
Hospital or institution stay	300	43/185 (23.24%)	33/115 (28.70%)	0.752 (0.443–1.277)	0.292	0.736 (0.419–1.293)	0.286	0.736 (0.418–1.296)	0.289
Dementia	302	67/187 (35.83%)	26/115 (22.61%)	1.911 (1.126–3.245)	0.017	1.641 (0.909–2.962)	0.101	1.549 (0.853–2.812)	0.151
WORSE OUTCOME ^a^	336	156/210 (74.29%)	92/126 (73.02%)	1.068 (0.647–1.761)	0.798	0.912 (0.524–1.589)	0.745	0.871 (0.499–1.521)	0.627

^a^death *or* increase in mRS of ≥1 *or* hospital/institution stay *or* dementia

^b^adjusted for age, gender, years of education, CIRS score and NIHSS score

^c^adjusted for age, gender, years of education, CIRS score, NIHSS score and incidence of in-hospital delirium

**Table 6:** Influence of post-stroke transient cognitive impairment (CI) compared to ‘cognitively impaired’ patients on one-year prognosis

Variable	Data	Incidence, n (%)		Univariate regression model		Multivariate regression model^b^		Multivariate regression model^c^	
		Transient CI (***n*** = 234)	Cognitively impaired (***n*** = 78)	OR (95%CI)	***P***-value	OR (95%CI)	***P***-value	OR (95%CI)	***P***-value
Mortality	287	21/215 (9.77%)	16/72 (22.22%)	0.379 (0.185–0.775)	0.008	0.465 (0.209–1.035)	0.061	0.521 (0.228–1.188)	0.121
Increase in mRS of ≥1	236	43/185 (23.24%)	11/51 (21.57%)	0.709 (0.391–1.284)	0.257	0.798 (0.420–1.516)	0.491	0.830 (0.434–1.588)	0.573
Hospital or institution stay	236	43/185 (23.24%)	11/51 (21.57%)	1.101 (0.520–2.330)	0.801	1.050 (0.281–2.294)	0.902	1.065 (0.486–2.334)	0.875
Dementia	241	67/187 (35.83%)	21/54 (38.89%)	0.877 (0.470–1.637)	0.681	1.131 (0.553–2.310)	0.736	1.246 (0.593–2.616)	0.561
WORSE OUTCOME ^a^	282	156/210 (74.29%)	55/72 (76.39%)	0.893 (0.478–1.669)	0.723	1.211 (0.610–2.404)	0.585	1.336 (0.663–2.692)	0.418

^a^death *or* increase in mRS of ≥1 *or* hospital/institution stay *or* dementia

^b^adjusted for age, gender, years of education, CIRS score and NIHSS score

^c^adjusted for age, gender, years of education, CIRS score, NIHSS score and incidence of in-hospital delirium

We also performed a post-hoc analysis, where the effect of transient CI on prognosis was calculated after excluding cases with delirium. The results are presented in the supplementary material (Tables S2, S3, S4, S5). Similarly, the only significant association in multivariate regression models was a lower risk of hospital or nursing home stay in patients with transient CI 3 months after stroke compared to “cognitively impaired” individuals (OR 0.473, 95%CI 0.232–0.946, *p* = 0.039).

In the second post-hoc analysis, our study population was divided into two subgroups based on the first MoCA score with a cut-off point between 23 and 24. The influence of transient CI on prognosis is shown in the supplementary material (Tables S6, S7, S8, S9, S10, S11, S12 and S13). We did not observe many differences in the results comparing to the main analysis. Based on the multivariate regression model, in the subgroup of cases with first MoCA score ≥ 24 patients with transient CI had a lower risk for the worse outcome 3 months after stroke compared to “cognitively impaired” (OR 0.279, 95%CI 0.082–0.948, *p* = 0.041), while in the subgroup of cases with first MoCA score < 24 patients with transient CI had a lower risk for the worse outcome 3 months after stroke compared to “cognitively stable” (OR 0.151, 95%CI 0.034–0.659, *p* = 0.012).

## Discussion

The results of the study showed that transient CI was a common complication in the acute phase of stroke, affecting about half of the patients studied. Transient CI was significantly associated with the occurrence of delirium. The presence of transient CI had no negative impact on long-term prognosis in both the overall analysis and additional analyses with cognitive impairment severity (≥24 vs < 24 points in MoCA). Patients with transient CI were less likely to require nursing home placement at 3 months after stroke than patients with permanent cognitive impairment.

The prevalence of transient CI in our study was 52.35%. In the only study published to date on transient CI after stroke, Pendlebury et al. assessed the prevalence of transient CI after stroke, defining transient CI as a baseline Mini-Mental State Examination (MMSE) score ≥ 2 points lower than at follow-up 1 month later [8]. The prevalence of transient CI was 38.9 and 19% depending on the time at which the baseline assessment was performed: within the first 7 days and 7 days after the event, respectively. Patients were included in this study after TIA or minor stroke. We decided to use the MoCA as a more appropriate test for assessing cognitive deficits in patients with vascular cognitive impairment than MMSE, and to evaluate patients for transient CI only in the acute phase of stroke. Currently, there is no established definition of transient CI after stroke. However, the problem appears to be frequent and relevant in clinical practice, and efforts should be made to develop one.

Our study showed no effect of transient CI in the acute phase of stroke on one-year prognosis compared to patients with stable cognition. Compared to “cognitively impaired” patients, those with transient CI had a lower risk of hospital or institution stay 3 months after stroke. No other significant associations were found, including mortality, disability and dementia risk. Pendlebury et al. showed that transient CI after TIA or minor stroke was associated with subsequent cognitive long-term cognitive decline. The correlation between transient CI and mortality, disability or institutionalization was not assessed in this study [8].

In our study, only the change in MoCA score during the hospital stay was considered, regardless of the patient’s cognitive status. However, cognitive deficits have been shown to affect poorer prognosis [24]. Patients with transient CI and stable during hospitalization did not differ in IQCODE questionnaire scores at admission. To get a better look at the possible effect of cognitive performance level on prognosis, we divided patients into subgroups according to their first MoCA score. Patients with a higher MoCA score (≥ 24) and transient CI had a better prognosis compared to those with declining cognition, while patients with a lower MoCA (< 24) score and transient CI had a better prognosis compared to those with stable cognition. No significant differences were found when considering mortality, disability, institutionalization and incidence of long-term dementia separately.

Cognitive decline early after stroke adversely affects prognosis. Zietemann et al. confirmed that decreased MoCA score during first 7 days after stroke correlated with an increased risk of three-year mortality, functional impairment and cognitive impairment [25], whereas Li et al. found that cognitive impairment diagnosed within 7 days after stroke was associated with higher risk of disability and poor activity of daily living in six-month follow-up [5]. These observations suggest that transient CI in the acute phase of stroke may not be as detrimental as persistent cognitive impairment and that the prognosis for patients with transient CI is more similar to those without cognitive deficits after stroke. Transient CI probably reflects a transient disturbance of brain homeostasis after stroke, rather than a trigger mechanism of the dementing process. This issue requires further research.

The relationship between delirium and transient CI has not been studied to date. Delirium is a complex condition that most commonly affects older patients in acute hospital settings. The definition of delirium includes transient and fluctuating symptoms of impaired attention and consciousness, as well as CI such as memory or language deficits [26]. In our study delirium was diagnosed in only 17.52% of patients with transient CI, and it was found to be an independent risk factor for transient CI in the acute phase of stroke. Also Pendlebury et al. described a possible role of delirium in the development of transient CI [8]. Patients with transient CI and patients with delirium have different long-term prognosis. Unlike patients with transient CI, patients with delirium in the acute phase of stroke have negative prognosis [12]. Although some features of delirium and transient CI may overlap, delirium and transient CI are two different disease entities, and although there appears to be an association between delirium and transient CI, the mechanism and underlying factors remain unclear and require further study.

Recent studies link the MoCA to the length of education time [27]. Polish patients are generally characterized by a long duration of education in the community. Patients included in the study had an average history of 11–12 years of education, which may explain the relatively high MoCA score in this population and confirm previous observations.

The results of our study may constitute the base for further research. The problem of transient CI after stroke cannot be overlooked in the clinical practice, as its prevalence seems to be high. However, there is still no accurate definition of transient CI. The use of cognitive assessment screening scales, should be extended with full neuropsychological examination. Prospective studies might find most effective method for transient CI diagnosis. Furthermore, more emphasis should be put on the differentiation between transient CI, delirium and permanent cognitive deficits. These three conditions seem to have separate underlying mechanism, and have different influence for further patient’s condition and prognosis.

Our study has several advantages including large baseline study population, prospective design and complex evaluation of neurocognitive status in hospital. The same trained psychologist was responsible for all cognitive assessments making the bias of interobserver variation minimal. Moreover, the examination was supervised and all data were evaluated by the senior neurologist/neuropsychologist. To determine CI before stroke, information on cognitive status was obtained from caregivers on admission using the IQCODE. Scores on this scale did not differ between patients with fluctuating CI and others, nor was it a risk factor for transient CI during hospital stay. Presence of delirium was carefully evaluated every day.

We can find limitations in this research. First, we had to excluded approximately 40% of patients from the original pre-qualified group. The predominant reasons for exclusion from the study were factors preventing MoCA testing, such as aphasia, visual impairment or in-hospital death. Second, we listed a few differences between included and excluded cases that could influence the results and constituted bias. Third, although we used MoCA, two parallel versions to asses change in cognitive functioning, the difference of 2 points between two measurements and the time of measurement were arbitrarily agreed upon. Also, most of the study participants were not under constant neurologic care in our center after hospital discharge, so the study population could differ in the level of post-stroke rehabilitation plan, secondary prevention and treatment of stroke complication when examined at the follow-up time points.

## Conclusions

In conclusion, transient CI, which often occurs in the acute phase of stroke, does not increase the risk of long-term complications, regardless of the level of cognitive functioning in the acute stage of stroke. Differentiation between transient CI, delirium and permanent cognitive deficits seems important in clinical practice for proper assessment of long-term prognosis.

## Supplementary Information

**Additional file 1: Table S1.** Comparison of patients with transient cognitive impairment (CI), stable MoCA score and cognitively impaired. **Table S2.** Influence of post-stroke transient cognitive impairment (CI) compared to ‘cognitively stable’ patients on three-month prognosis. Patients with delirium are excluded from the analysis. **Table S3.** Influence of post-stroke transient cognitive impairment (CI) compared to ‘cognitively impaired’ patients on three-month prognosis. Patients with delirium are excluded from the analysis. **Table S4.** Influence of post-stroke transient cognitive impairment (CI) compared to ‘cognitively stable’ patients on one-year prognosis. Patients with delirium are excluded from the analysis. **Table S5.** Influence of post-stroke transient cognitive impairment (CI) compared to ‘cognitively impaired’ patients on one-year prognosis. Patients with delirium are excluded from the analysis. **Table S6.** Influence of post-stroke transient cognitive impairment (CI) compared to ‘cognitively stable’ patients on three-month prognosis. Only patients with first MoCA score ≥ 24 are included. **Table S7.** Influence of post-stroke transient cognitive impairment (CI) compared to ‘cognitively impaired’ patients on three-month prognosis. Only patients with first MoCA score ≥ 24 are included. **Table S8.** Influence of post-stroke transient cognitive impairment (CI) compared to ‘cognitively stable’ patients on one-year prognosis. Only patients with first MoCA score ≥ 24 are included. **Table S9.** Influence of post-stroke transient cognitive impairment (CI) compared to ‘cognitively impaired’ patients on one-year prognosis. Only patients with first MoCA score ≥ 24 are included. **Table S10.** Influence of post-stroke transient cognitive impairment (CI) compared to ‘cognitively stable’ patients on three-month prognosis. Only patients with first MoCA score < 23 are included. **Table S11.** Influence of post-stroke transient cognitive impairment (CI) compared to ‘cognitively impaired’ patients on three-month prognosis. Only patients with first MoCA score < 23 are included. **Table S12.** Influence of post-stroke transient cognitive impairment (CI) compared to ‘cognitively stable’ patients on one-year prognosis. Only patients with first MoCA score < 23 are included. **Table S13.** Influence of post-stroke transient cognitive impairment (CI) compared to ‘cognitively impaired’ patients on one-year prognosis. Only patients with first MoCA score < 23 are included.

## Data Availability

The datasets used and/or analyzed during the current study available from the corresponding author on reasonable request.
