# Supplementary material for: Transient cognitive impairment in the acute phase of stroke – prevalence, risk factors and influence on long-term prognosis in population of patients with stroke (research study – part of the PROPOLIS study)
Source: BMC Neurol. 2023 Feb 17;23:75. doi: 10.1186/s12883-023-03120-x (PMC9936649; doi:10.1186/s12883-023-03120-x)
Supplement: Supplementary file 1 — Additional file 1: Table S1. Comparison of patients with transient cognitive impairment (CI), stable MoCA score and cognitively impaired. Table S2. Influence of post-stroke transient cognitive impairment (CI) compared to ‘cognitively stable’ patients on three-month prognosis. Patients with delirium are excluded from the analysis. Table S3. Influence of post-stroke transient cognitive impairment (CI) compared to ‘cognitively impaired’ patients on three-month prognosis. Patients with delirium are excluded from the analysis. Table S4. Influence of post-stroke transient cognitive impairment (CI) compared to ‘cognitively stable’ patients on one-year prognosis. Patients with delirium are excluded from the analysis. Table S5. Influence of post-stroke transient cognitive impairment (CI) compared to ‘cognitively impaired’ patients on one-year prognosis. Patients with delirium are excluded from the analysis. Table S6. Influence of post-stroke transient cognitive impairment (CI) compared to ‘cognitively stable’ patients on three-month prognosis. Only patients with first MoCA score ≥ 24 are included. Table S7. Influence of post-stroke transient cognitive impairment (CI) compared to ‘cognitively impaired’ patients on three-month prognosis. Only patients with first MoCA score ≥ 24 are included. Table S8. Influence of post-stroke transient cognitive impairment (CI) compared to ‘cognitively stable’ patients on one-year prognosis. Only patients with first MoCA score ≥ 24 are included. Table S9. Influence of post-stroke transient cognitive impairment (CI) compared to ‘cognitively impaired’ patients on one-year prognosis. Only patients with first MoCA score ≥ 24 are included. Table S10. Influence of post-stroke transient cognitive impairment (CI) compared to ‘cognitively stable’ patients on three-month prognosis. Only patients with first MoCA score < 23 are included. Table S11. Influence of post-stroke transient cognitive impairment (CI) compared to ‘cognitively impaired’ patients on [file 12883_2023_3120_MOESM1_ESM.docx]

**Table S1.** Comparison of patients with transient cognitive impairment (CI), stable MoCA score and cognitively impaired.

| **VARIABLE** | **Data** | **TRANSIENT CI**  **(n=234)** | **COGNITIVELY STABLE (n=135)** | **COGNITIVELY IMPAIRED (n=78)** | **P-value** |
| --- | --- | --- | --- | --- | --- |
|  |  |  |  |  |  |
| **First MoCA score** ‡ | 447 | **19 (13-23)** | **24 (19-26)** | **20 (15-25)** | <0.001 |
| **Second MoCA score** ‡ | 447 | **24 (20-27)** | **23 (19-27)** | **17 (10-22)** | <0.001 |
|  |  |  |  |  |  |
| Male gender † | 447 | 124/234 (52.99%) | 73/135 (54.07%) | 33/78 (42.31%) | 0.200 |
| Age [years] ‡ | 447 | 68.5 (62-79) | 65 (58-77) | 75 (65-82) | <0.001 |
| BMI [kg/m^2^] ‡ | 437 | 26.73 (24.07-30.08) | 25.78 (23.44-29.38) | 27.38 (23.53-30.84) | 0.403 |
| Length of education [years] ‡ | 438 | 11 (8-13) | 12 (10-14) | 11 (9-12) | 0.013 |
|  |  |  |  |  |  |
| Hemorrhagic stroke † | 447 | 14/234 (5.98%) | 8/135 (5.93%) | 6/78 (7.69%) | 0.855 |
| TOAST classification |  |  |  |  |  |
| - large-artery atherosclerosis † | 401 | 24/211 (11.37%) | 10/121 (8.26%) | 7/69 (10.14%) | 0.659 |
| - cardioembolism † | 401 | 12/211 (5.69%) | 11/121 (9.09%) | 1/69 (1.45%) | 0.067 |
| - small-vessel occlusion † | 401 | 66/211 (32.28%) | 33/121 (27.27%) | 26/69 (37.68%) | 0.333 |
| - other determined etiology † | 401 | 109/211 (51.66%) | 64/121 (52.89%) | 34/69 (49.28%) | 0.891 |
| - undetermined etiology † | 401 | 0/211 (0%) | 3/121 (2.48%) | 1/69 (1.45%) | 0.044 |
| Side of stroke |  |  |  |  |  |
| - right hemisphere † | 447 | 106/234 (45.30%) | 67/135 (49.63%) | 35/78 (44.87%) | 0.688 |
| - left hemisphere † | 447 | 94/234 (40.17%) | 48/135 (35.56%) | 28/78 (35.90%) | 0.620 |
| - posterior part † | 447 | 30/234 (12.82%) | 19/135 (14.07%) | 12/78 (15.38%) | 0.839 |
| - more than one localization † | 447 | 4/234 (1.71%) | 1/135 (0.74%) | 3/78 (3.85%) | 0.285 |
| rt-Pa treatment † | 447 | 62/234 (26.50%) | 24/135 (17.78%) | 22/78 (28.21%) | 0.102 |
| Thrombectomy † | 447 | 10/234 (4.27%) | 5/135 (3.70%) | 4/78 (5.13%) | 0.886 |
|  |  |  |  |  |  |
| Medical history |  |  |  |  |  |
| - hypertension † | 447 | 162/234 (69.23%) | 92/135 (68.15%) | 57/78 (73.08%) | 0.739 |
| - diabetes † | 447 | 66/234 (28.21%) | 36/135 (26.67%) | 22/78 (28.21%) | 0.946 |
| - atrial fibrillation † | 447 | 50/234 (21.37%) | 18/135 (13.33%) | 21/78 (26.92%) | 0.038 |
| - myocardial infraction † | 447 | 30/234 (21.82%) | 18/135 (13.33%) | 10/78 (12.82%) | 0.989 |
| - PCI or CABG † | 447 | 16/234 (6.84%) | 12/135 (8.89%) | 7/78 (8.97%) | 0.715 |
| - smoking – ever † | 446 | 18/233 (50.64%) | 73/135 (54.07%) | 36/78 (46.15%) | 0.534 |
| - previous stroke or TIA † | 445 | 41/233 (17.60%) | 24/134 (17.91%) | 16/78 (20.51%) | 0.845 |
| CIRS, total score ‡ | 447 | 7 (5-11) | 8 (4-12) | 10 (7-13) | 0.014 |
|  |  |  |  |  |  |
| Aphasia in hospital † | 447 | 35/234 (14.96%) | 14/135 (10.37%) | 14/78 (17.95%) | 0.258 |
| Neglect in hospital † | 447 | 22/234 (9.40%) | 15/135 (11.11%) | 17/78 (21.79%) | 0.022 |
| Vision deficits in hospital † | 447 | 64/234 (27.35%) | 29/135 (21.48%) | 30/78 (38.46%) | 0.031 |
| Delirium in hospital † | 447 | 41/234 (17.52%) | 10/135 (7.41%) | 26/78 (33.33%) | <0.001 |
|  |  |  |  |  |  |
| NIHSS at admission ‡ | 447 | 4 (2-9) | 3 (2-7) | 4.5 (2-11) | 0.133 |
| Pre-hospital mRS ‡ | 447 | 0 (0-0) | 0 (0-0) | 0 (0-1) | 0.025 |
| Pre-hospital IQCODE ‡ | 378 | 78 (78-82) | 78 (78-80) | 78 (78-82) | 0.113 |
|  |  |  |  |  |  |
| CRP level in hospital [mg/l] ‡ | 432 | 4.12 (1.88-11.40) | 4.57 (1.78-11.96) | 8.59 (2.97-17.72) | 0.008 |
|  |  |  |  |  |  |

† n (%) ‡ median (IQR)

**Table S2.** Influence of post-stroke transient cognitive impairment (CI) compared to ‘cognitively stable’ patients on three-month prognosis. Patients with delirium are excluded from the analysis

|  |  | INCIDENCE, n (%) | | UNIVARIATE REGRESSION MODEL | | MULTIVARIATE REGRESSION MODEL ^1^ | |
| --- | --- | --- | --- | --- | --- | --- | --- |
| **VARIABLE** | **Data** | **TRANSIENT CI**  **(n=193)** | **COGNITIVELY STABLE (n=125)** | **OR (95%CI)** | **P-value** | **OR (95%CI)** | **P-value** |
|  |  |  |  |  |  |  |  |
| Mortality | 314 | 6/192 (3.13%) | 2/122 (1.64%) | 1.935  (0.384-9.748) | 0.423 | 1.915  (0.359-10.208) | 0.269 |
| Increase in mRS of ≥1 | 303 | 102/186 (54.84%) | 71/117 (60.68%) | 0.787  (0.492-1.259) | 0.317 | 0.676  (0.409-1.117) | 0.126 |
| Hospital or institution stay | 304 | 41/187 (21.93%) | 27/117 (23.08%) | 0.936  (0.539-1.626) | 0.814 | 0.927  (0.521-1.649) | 0.796 |
| Dementia | 236 | 29/148 (19.59%) | 17/88 (19.32%) | 1.018  (0.522-1.983) | 0.959 | 0.908  (0.429-1.918) | 0.800 |
| WORSE OUTCOME § | 275 | 117/170 (68.82%) | 79/105 (75.24%) | 0.727  (0.419-1.258) | 0.254 | 0.580  (0.320-1.050) | 0.072 |
|  |  |  |  |  |  |  |  |

§ death *or* increase in mRS of ≥1 *or* hospital/institution stay *or* dementia

^1^ adjusted for age, gender, years of education, CIRS score and NIHSS score

**Table S3.** Influence of post-stroke transient cognitive impairment (CI) compared to ‘cognitively impaired’ patients on three-month prognosis. Patients with delirium are excluded from the analysis

|  |  | INCIDENCE, n (%) | | UNIVARIATE REGRESSION MODEL | | MULTIVARIATE REGRESSION MODEL ^1^ | |
| --- | --- | --- | --- | --- | --- | --- | --- |
| **VARIABLE** | **Data** | **TRANSIENT CI**  **(n=193)** | **COGNITIVELY IMPAIRED (n=52)** | **OR (95%CI)** | **P-value** | **OR (95%CI)** | **P-value** |
|  |  |  |  |  |  |  |  |
| Mortality | 243 | 6/192 (3.13%) | 2/51 (3.92%) | 0.790  (0.155-4.038) | 0.777 | 1.302  (0.216-7.838) | 0.773 |
| Increase in mRS of ≥1 | 234 | 102/186 (54.84%) | 33/48 (68.75%) | 0.552  (0.281-1.084) | 0.085 | 0.619  (0.308-1.246) | 0.179 |
| Hospital or institution stay | 235 | 41/187 (21.93%) | 17/48 (35.42%) | 0.512  (0.258-1.016) | 0.056 | 0.473  (0.232-0.946) | 0.039 |
| Dementia | 186 | 29/148 (19.59%) | 7/38 (18.42%) | 1.079  (0.432-2.695) | 0.870 | 1.266  (0.480-3.338) | 0.633 |
| WORSE OUTCOME § | 215 | 117/170 (68.82%) | 37/45 (82.22%) | 0.477  (0.208-1.095) | 0.081 | 0.523  (0.221-1.236) | 0.140 |
|  |  |  |  |  |  |  |  |

§ death *or* increase in mRS of ≥1 *or* hospital/institution stay *or* dementia

^1^ adjusted for age, gender, years of education, CIRS score and NIHSS score

**Table S4.** Influence of post-stroke transient cognitive impairment (CI) compared to ‘cognitively stable’ patients on one-year prognosis. Patients with delirium are excluded from the analysis

|  |  | INCIDENCE, n (%) | | UNIVARIATE REGRESSION MODEL | | MULTIVARIATE REGRESSION MODEL ^1^ | |
| --- | --- | --- | --- | --- | --- | --- | --- |
| **VARIABLE** | **Data** | **TRANSIENT CI**  **(n=193)** | **COGNITIVELY STABLE (n=125)** | **OR (95%CI)** | **P-value** | **OR (95%CI)** | **P-value** |
|  |  |  |  |  |  |  |  |
| Mortality | 299 | 12/180 (6.67%) | 6/119 (5.04%) | 1.345  (0.491-3.688) | 0.564 | 1.238  (0.426-3.592) | 0.695 |
| Increase in mRS of ≥1 | 291 | 93/174 (53.45%) | 64/117 (54.70%) | 0.951  (0.594-1.522) | 0.834 | 0.891  (0.542-1.466) | 0.650 |
| Hospital or institution stay | 269 | 36/161 (22.36%) | 30/108 (27.78%) | 0.749  (0.427-1.312) | 0.312 | 0.728  (0.400-1.323) | 0.298 |
| Dementia | 270 | 51/161 (31.68%) | 23/109 (21.10%) | 1.734  (0.983-3.058) | 0.057 | 1.586  (0.843-2.983) | 0.152 |
| WORSE OUTCOME § | 292 | 124/176 (70.45%) | 83/116 (71.55%) | 0.948  (0.565-1.590) | 0.840 | 0.843  (0.478-1.488) | 0.556 |
|  |  |  |  |  |  |  |  |

§ death *or* increase in mRS of ≥1 *or* hospital/institution stay *or* dementia

^1^ adjusted for age, gender, years of education, CIRS score and NIHSS score

**Table S5.** Influence of post-stroke transient cognitive impairment (CI) compared to ‘cognitively impaired’ patients on one-year prognosis. Patients with delirium are excluded from the analysis

|  |  | INCIDENCE, n (%) | | UNIVARIATE REGRESSION MODEL | | MULTIVARIATE REGRESSION MODEL ^1^ | |
| --- | --- | --- | --- | --- | --- | --- | --- |
| **VARIABLE** | **Data** | **TRANSIENT CI**  **(n=193)** | **COGNITIVELY IMPAIRED (n=52)** | **OR (95%CI)** | **P-value** | **OR (95%CI)** | **P-value** |
|  |  |  |  |  |  |  |  |
| Mortality | 229 | 12/180 (6.67%) | 5/49 (10.20%) | 0.629  (0.210-1.879) | 0.406 | 0.726  (0.222-2.369) | 0.595 |
| Increase in mRS of ≥1 | 221 | 93/174 (53.45%) | 28/47 (59.57%) | 0.779  (0.405-1.499) | 0.455 | 0.827  (0.415-1.647) | 0.589 |
| Hospital or institution stay | 202 | 36/161 (22.36%) | 7/41 (17.07%) | 1.399  (0.572-3.420) | 0.462 | 1.421  (0.563-3.584) | 0.457 |
| Dementia | 204 | 51/161 (31.68%) | 11/43 (25.58%) | 1.349  (0.630-2.888) | 0.441 | 1.862  (0.784-4.422) | 0.159 |
| WORSE OUTCOME § | 225 | 124/176 (70.45%) | 33/49 (67.35%) | 1.156  (0.586-2.280) | 0.675 | 1.397  (0.676-2.886) | 0.366 |
|  |  |  |  |  |  |  |  |

§ death *or* increase in mRS of ≥1 *or* hospital/institution stay *or* dementia

^1^ adjusted for age, gender, years of education, CIRS score and NIHSS score

**Table S6.** Influence of post-stroke transient cognitive impairment (CI) compared to ‘cognitively stable’ patients on three-month prognosis. Only patients with first MoCA score ≥ 24 are included

|  |  | INCIDENCE, n (%) | | UNIVARIATE REGRESSION MODEL | | MULTIVARIATE REGRESSION MODEL ^1^ | |
| --- | --- | --- | --- | --- | --- | --- | --- |
| **VARIABLE** | **Data** | **TRANSIENT CI**  **(n=46)** | **COGNITIVELY STABLE (n=69)** | **OR (95%CI)** | **P-value** | **OR (95%CI)** | **P-value** |
|  |  |  |  |  |  |  |  |
| Mortality | 113 | 1/46 (3.13%) | 0/67 (0%) | N/A | N/A | N/A | N/A |
| Increase in mRS of ≥1 | 108 | 11/42 (26.19%) | 31/66 (36.97%) | 0.401  (0.173-0.929) | 0.033 | 0.458  (0.185-1.134) | 0.091 |
| Hospital or institution stay | 108 | 4/42 (9.52%) | 9/66 (13.64%) | 0.667  (0.192-2.321) | 0.524 | 0.808  (0.216-3.023) | 0.751 |
| Dementia | 84 | 2/33 (6.06%) | 1/51 (1.96%) | 3.266  (0.281-37.082) | 0.347 | 2.998  (0.252-35.717) | 0.385 |
| WORSE OUTCOME § | 92 | 13/36 (36.11%) | 32/56 (57.14%) | 0.424  (0.179-1.004) | 0.051 | 0.464  (0.184-1.167) | 0.103 |
|  |  |  |  |  |  |  |  |

§ death *or* increase in mRS of ≥1 *or* hospital/institution stay *or* dementia

^1^ adjusted for age, gender, years of education, CIRS score and NIHSS score

**Table S7.** Influence of post-stroke transient cognitive impairment (CI) compared to ‘cognitively impaired’ patients on three-month prognosis. Only patients with first MoCA score ≥ 24 are included

|  |  | INCIDENCE, n (%) | | UNIVARIATE REGRESSION MODEL | | MULTIVARIATE REGRESSION MODEL ^1^ | |
| --- | --- | --- | --- | --- | --- | --- | --- |
| **VARIABLE** | **Data** | **TRANSIENT CI**  **(n=46)** | **COGNITIVELY IMPAIRED (n=29)** | **OR (95%CI)** | **P-value** | **OR (95%CI)** | **P-value** |
|  |  |  |  |  |  |  |  |
| Mortality | 74 | 1/46 (3.13%) | 1/28 (3.57%) | 0.600  (0.036-9.992) | 0.772 | 1.636  (0.053-50.795) | 0.779 |
| Increase in mRS of ≥1 | 69 | 11/42 (26.19%) | 15/27 (55.56%) | 0.284  (0.102-0.791) | 0.016 | 0.427  (0.131-1.387) | 0.157 |
| Hospital or institution stay | 69 | 4/42 (9.52%) | 9/27 (33.33%) | 0.211  (0.057-0.776) | 0.019 | 0.164  (0.033-0.807) | 0.026 |
| Dementia | 56 | 2/33 (6.06%) | 0/23 (0.00%) | N/A | N/A | N/A | N/A |
| WORSE OUTCOME § | 61 | 13/36 (36.11%) | 18/25 (72.00%) | 0.220  (0.073-0.665) | 0.007 | 0.279  (0.082-0.948) | 0.041 |
|  |  |  |  |  |  |  |  |

§ death *or* increase in mRS of ≥1 *or* hospital/institution stay *or* dementia

^1^ adjusted for age, gender, years of education, CIRS score and NIHSS score

**Table S8.** Influence of post-stroke transient cognitive impairment (CI) compared to ‘cognitively stable’ patients on one-year prognosis. Only patients with first MoCA score ≥ 24 are included

|  |  | INCIDENCE, n (%) | | UNIVARIATE REGRESSION MODEL | | MULTIVARIATE REGRESSION MODEL ^1^ | |
| --- | --- | --- | --- | --- | --- | --- | --- |
| **VARIABLE** | **Data** | **TRANSIENT CI**  **(n=46)** | **COGNITIVELY STABLE (n=69)** | **OR (95%CI)** | **P-value** | **OR (95%CI)** | **P-value** |
|  |  |  |  |  |  |  |  |
| Mortality | 109 | 1/44 (2.27%) | 0/65 (0%) | N/A | N/A | N/A | N/A |
| Increase in mRS of ≥1 | 108 | 16/43 (37.21%) | 29/65 (44.62%) | 0.736  (0.334-1.618) | 0.445 | 0.917  (0.375-2.243) | 0.850 |
| Hospital or institution stay | 103 | 4/41 (9.76%) | 16/62 (25.81%) | 0.311  (0.096-1.010) | 0.052 | 0.443  (0.127-1.549) | 0.203 |
| Dementia | 106 | 2/42 (4.76%) | 5/64 (7.81%) | 0.590  (0.109-3.191) | 0.540 | 0.617  (0.096-3.975) | 0.612 |
| WORSE OUTCOME § | 103 | 20/41 (48.78%) | 38/62 (61.29%) | 0.602  (0.271-1.335) | 0.212 | 0.753  (0.304-1.869) | 0.541 |
|  |  |  |  |  |  |  |  |

§ death *or* increase in mRS of ≥1 *or* hospital/institution stay *or* dementia

^1^ adjusted for age, gender, years of education, CIRS score and NIHSS score

**Table S9.** Influence of post-stroke transient cognitive impairment (CI) compared to ‘cognitively impaired’ patients on one-year prognosis. Only patients with first MoCA score ≥ 24 are included

|  |  | INCIDENCE, n (%) | | UNIVARIATE REGRESSION MODEL | | MULTIVARIATE REGRESSION MODEL ^1^ | |
| --- | --- | --- | --- | --- | --- | --- | --- |
| **VARIABLE** | **Data** | **TRANSIENT CI**  **(n=46)** | **COGNITIVELY IMPAIRED (n=29)** | **OR (95%CI)** | **P-value** | **OR (95%CI)** | **P-value** |
|  |  |  |  |  |  |  |  |
| Mortality | 72 | 1/44 (2.27%) | 2/28 (7.14%) | 0.302  (0.026-3.501) | 0.338 | 0.446  (0.023-8.663) | 0.593 |
| Increase in mRS of ≥1 | 70 | 16/43 (37.21%) | 14/27 (51.85%) | 0.550  (0.207-1.460) | 0.230 | 0.876  (0.288-2.668) | 0.816 |
| Hospital or institution stay | 66 | 4/41 (9.76%) | 4/25 (16.00%) | 0.568  (0.128-2.508) | 0.455 | 1.021  (0.186-5.601) | 0.981 |
| Dementia | 68 | 2/42 (4.76%) | 2/26 (7.69%) | 0.600  (0.079-4.542) | 0.621 | 0.464  (0.045-4.831) | 0.521 |
| WORSE OUTCOME § | 69 | 20/41 (48.78%) | 16/28 (57.14%) | 0.714  (0.272-1.879) | 0.495 | 1.157  (0.388-3.454) | 0.794 |
|  |  |  |  |  |  |  |  |

§ death *or* increase in mRS of ≥1 *or* hospital/institution stay *or* dementia

^1^ adjusted for age, gender, years of education, CIRS score and NIHSS score

**Table S10.** Influence of post-stroke transient cognitive impairment (CI) compared to ‘cognitively stable’ patients on three-month prognosis. Only patients with first MoCA score < 23 are included

|  |  | INCIDENCE, n (%) | | UNIVARIATE REGRESSION MODEL | | MULTIVARIATE REGRESSION MODEL ^1^ | |
| --- | --- | --- | --- | --- | --- | --- | --- |
| **VARIABLE** | **Data** | **TRANSIENT CI**  **(n=188)** | **COGNITIVELY STABLE (n=66)** | **OR (95%CI)** | **P-value** | **OR (95%CI)** | **P-value** |
|  |  |  |  |  |  |  |  |
| Mortality | 250 | 9/185 (4.86%) | 3/65 (4.62%) | 1.057  (0.277-4.029) | 0.936 | 1.202  (0.291-4.974) | 0.799 |
| Increase in mRS of ≥1 | 242 | 126/182 (69.23%) | 48/60 (80.00%) | 0.563  (0.277-1.140) | 0.111 | 0.590  (0.287-1.210) | 0.150 |
| Hospital or institution stay | 244 | 50/183 (27.32%) | 25/61 (40.98%) | 0.541  (0.296-0.991) | 0.047 | 0.544  (0.288-1.026) | 0.060 |
| Dementia | 186 | 43/141 (30.50%) | 20/45 (44.44%) | 0.548  (0.275-1.092) | 0.087 | 0.531  (0.249-1.133) | 0.102 |
| WORSE OUTCOME § | 231 | 141/172 (81.98%) | 57/59 (96.61%) | 0.160  (0.037-0.689) | 0.014 | 0.151  (0.034-0.659) | 0.012 |
|  |  |  |  |  |  |  |  |

§ death *or* increase in mRS of ≥1 *or* hospital/institution stay *or* dementia

^1^ adjusted for age, gender, years of education, CIRS score and NIHSS score

**Table S11.** Influence of post-stroke transient cognitive impairment (CI) compared to ‘cognitively impaired’ patients on three-month prognosis. Only patients with first MoCA score < 23 are included

|  |  | INCIDENCE, n (%) | | UNIVARIATE REGRESSION MODEL | | MULTIVARIATE REGRESSION MODEL ^1^ | |
| --- | --- | --- | --- | --- | --- | --- | --- |
| **VARIABLE** | **Data** | **TRANSIENT CI**  **(n=188)** | **COGNITIVELY IMPAIRED (n=49)** | **OR (95%CI)** | **P-value** | **OR (95%CI)** | **P-value** |
|  |  |  |  |  |  |  |  |
| Mortality | 232 | 9/185 (4.86%) | 9/47 (19.15%) | 0.216  (0.080-0.580) | 0.002 | 0.367  (0.124-1.082) | 0.069 |
| Increase in mRS of ≥1 | 225 | 126/182 (69.23%) | 38/43 (88.37%) | 0.296  (0.111-0.792) | 0.015 | 0.386  (0.139-1.072) | 0.068 |
| Hospital or institution stay | 226 | 50/183 (27.32%) | 22/43 (51.16%) | 0.359  (0.182-0.709) | 0.003 | 0.348  (0.161-0.755) | 0.008 |
| Dementia | 170 | 43/141 (30.50%) | 16/29 (55.17%) | 0.357  (0.158-0.805) | 0.013 | 0.497  (0.201-1.225) | 0.129 |
| WORSE OUTCOME § | 215 | 141/172 (81.98%) | 42/43 (97.67%) | 0.108  (0.014-0.817) | 0.031 | 0.147  (0.019-1.148) | 0.067 |
|  |  |  |  |  |  |  |  |

§ death *or* increase in mRS of ≥1 *or* hospital/institution stay *or* dementia

^1^ adjusted for age, gender, years of education, CIRS score and NIHSS score

**Table S12.** Influence of post-stroke transient cognitive impairment (CI) compared to ‘cognitively stable’ patients on one-year prognosis. Only patients with first MoCA score < 23 are included

|  |  | INCIDENCE, n (%) | | UNIVARIATE REGRESSION MODEL | | MULTIVARIATE REGRESSION MODEL ^1^ | |
| --- | --- | --- | --- | --- | --- | --- | --- |
| **VARIABLE** | **Data** | **TRANSIENT CI**  **(n=188)** | **COGNITIVELY STABLE (n=66)** | **OR (95%CI)** | **P-value** | **OR (95%CI)** | **P-value** |
|  |  |  |  |  |  |  |  |
| Mortality | 235 | 20/171 (11.70%) | 9/64 (14.06%) | 0.809  (0.348-1.885) | 0.624 | 0.882  (0.355-2.187) | 0.786 |
| Increase in mRS of ≥1 | 223 | 103/162 (63.58%) | 40/61 (65.57%) | 0.917  (0.494-1.700) | 0.782 | 1.030  (0.547-1.939) | 0.927 |
| Hospital or institution stay | 197 | 39/144 (27.08%) | 17/53 (32.08%) | 0.787  (0.397-1.559) | 0.491 | 0.817  (0.399-1.673) | 0.581 |
| Dementia | 196 | 65/145 (44.83%) | 21/51 (41.18%) | 1.161  (0.608-2.216) | 0.651 | 1.137  (0.566-2.284) | 0.717 |
| WORSE OUTCOME § | 233 | 136/169 (80.47%) | 54/64 (84.38%) | 0.763  (0.352-1.656) | 0.494 | 0.890  (0.399-1.987) | 0.777 |
|  |  |  |  |  |  |  |  |

§ death *or* increase in mRS of ≥1 *or* hospital/institution stay *or* dementia

^1^ adjusted for age, gender, years of education, CIRS score and NIHSS score

**Table S13.** Influence of post-stroke transient cognitive impairment (CI) compared to ‘cognitively impaired’ patients on one-year prognosis. Only patients with first MoCA score < 23 are included

|  |  | INCIDENCE, n (%) | | UNIVARIATE REGRESSION MODEL | | MULTIVARIATE REGRESSION MODEL ^1^ | |
| --- | --- | --- | --- | --- | --- | --- | --- |
| **VARIABLE** | **Data** | **TRANSIENT CI**  **(n=188)** | **COGNITIVELY IMPAIRED (n=49)** | **OR (95%CI)** | **P-value** | **OR (95%CI)** | **P-value** |
|  |  |  |  |  |  |  |  |
| Mortality | 215 | 20/171 (11.70%) | 14/44 (31.82%) | 0.284  (0.129-0.624) | 0.002 | 0.417  (0.173-1.009) | 0.052 |
| Increase in mRS of ≥1 | 197 | 103/162 (63.58%) | 27/35 (63.58%) | 0.517  (0.221-1.212) | 0.129 | 0.581  (0.225-1.500) | 0.262 |
| Hospital or institution stay | 170 | 39/144 (27.08%) | 7/26 (26.92%) | 1.008  (0.393-2.584) | 0.987 | 0.947  (0.341-2.634) | 0.918 |
| Dementia | 173 | 65/145 (44.83%) | 19/28 (67.86%) | 0.385  (0.163-0.908) | 0.029 | 0.576  (0.223-1.491) | 0.256 |
| WORSE OUTCOME § | 213 | 136/169 (80.47%) | 39/44 (88.64%) | 0.528  (0.193-1.444) | 0.214 | 0.747  (0.254-2.195) | 0.596 |
|  |  |  |  |  |  |  |  |

§ death *or* increase in mRS of ≥1 *or* hospital/institution stay *or* dementia

^1^ adjusted for age, gender, years of education, CIRS score and NIHSS score
